# Biosimilars in dermatology: New opportunities and obstacles

**DOI:** 10.1016/j.jdin.2025.05.003

**Published:** 2025-05-29

**Authors:** Melissa C. Leeolou, Justin L. Jia, Kavita Y. Sarin

**Affiliations:** Department of Dermatology, Stanford University School of Medicine, Stanford, California

**Keywords:** access to treatment, biologics, biosimilars, cost, drug pricing, health policy, health care reform, patient advocacy, specialty medication

Biologics have transformed the treatment landscape for dermatologic diseases. Early access to these therapies helps prevent complications associated with advanced disease and minimizes side effects linked to nonspecific immunomodulatory drugs. However, high costs often render them inaccessible. As patents for biologics expire, typically 20 years from the filing date, biosimilars—medications that are highly similar to biologics in both safety and efficacy—have emerged as a cost-effective alternative.^1^ Although biosimilars have the potential to lower costs and increase access, market dynamics and systemic barriers in the United States hinder their uptake.

Biosimilars have been validated through over a decade of global use, amounting to 700 million patient-treatment days.^2^ Although originator biologics require extensive research from drug discovery through clinical trials, biosimilars demonstrate similarity to their reference biologic. In 2018, the US Food and Drug Administration (FDA) introduced the Biosimilars Action Plan to increase biosimilar entry into the US market by improving approval efficiencies and clarifying regulatory processes.^2^

Despite demonstrated efficacy and streamlined FDA approvals, biosimilars have been delayed by patent disputes. Using the FDAs publicly available databases, we found that adalimumab-atto, a biosimilar to adalimumab, was FDA-approved in September 2016 but became commercially available more than 6 years later, in January 2023 (Table I).^3^ Of the 16 biosimilars with dermatological indications commercially available in the United States, the median time from approval to commercial availability was 12.5 months (range: 0-76).

**Table I:** US regulatory approval and commercial availability dates for biosimilars with dermatological indications^3^

Medication trade name (generic name)	Approval date∗	Availability date†	Dermatological indications
Humira (adalimumab)			Hidradenitis suppurativa, psoriasis
Amjevita (adalimumab-atto)	September 2016	January 2023	
Cyltezo (adalimumab-adbm)	August 2017	July 2023	
Hyrimoz (adalimumab-adaz)	October 2018	July 2023	
Hadlima (adalimumab-bwwd)	July 2019	July 2023	
Abrilada (adalimumab-afzb)	November 2019	October 2023	
Hulio (adalimumab-fkjp)	July 2020	July 2023	
Yusimry (adalimumab-aqvh)	December 2021	July 2023	
Idacio (adalimumab-aacf)	December 2022	July 2023	
Yuflyma (adalimumab-aaty)	May 2023	May 2023	
Simlandi (adalimumab-ryvk)	February 2024	May 2024	
Enbrel (etanercept)			Psoriasis
Erelzi (etanercept-szzs)	August 2016		
Eticovo (etanercept-ykro)	April 2019		
Remicade (infliximab)			Psoriasis
Inflectra (infliximab-dyyb)	April 2016	November 2016	
Renflexis (infliximab-abda)	May 2017	July 2017	
Ixifi (infliximab-qbtx)	December 2017		
Avsola (infliximab-axxq)	December 2019	December 2019	
Rituxan (rituximab)			Pemphigus vulgaris
Truxima (rituximab-abbs)	November 2018	May 2020	
Ruxience (rituximab-pvvr)	July 2019	January 2020	
Riabni (rituximab-arrx)	December 2020	January 2021	
Stelara (ustekinumab)			Psoriasis
Wezlana (ustekinumab-auub)	October 2023		
Selarsdi (ustekinumab-aekn)	April 2024		
Pyzchiva (ustekinumab-ttwe)	June 2024		
Otulfi (ustekinumab-aauz)	September 2024		
Imuldosa (ustekinumab-srlf)	October 2024		

∗ US Food and Drug Administration approval date.

† US Food and Drug Administration marketing start date. If blank, marketing has not started as of manuscript submission date.

In addition to patent disputes, practices by pharmacy benefit managers and insurers may hinder access. Biologics still receive preferential placement on formularies because pharmacy benefit managers may negotiate larger rebates with manufacturers, some of which may be shared with insurers. Biosimilars generally cost 15% to 30% less than their reference biologic, but these savings might not outweigh rebate gains.^4^

Systemic reforms are needed to realize the potential of biosimilars. Several federal bills have been proposed to address systemic barriers that deter biosimilars from reaching the market. For example, federal US Senate bill 142 (S.142), introduced in 2023, would prohibit brand-name drug companies from compensating biosimilar manufacturers to delay entry into the market, dismantling practices that limit access to affordable alternatives.^5^ Although these efforts aim to reduce delays in market entry, the reality is more nuanced. Many biosimilar manufacturers are the same companies producing originator biologics or subsidiaries of originators, complicating efforts to foster market competition.

Further reforms at multiple levels are needed. Fair competition in the biosimilar market requires pharmacy benefit manager transparency to eliminate financial barriers alongside policies that prevent excessive patent protections. Simplified insurance processes are needed to reduce administrative challenges for smaller practices. Finally, reducing manufacturing costs through policy support could lower barriers for new manufacturers, promoting broader market participation.

Biosimilars offer an opportunity to improve access to treatment, but barriers have kept this potential out of reach. Delays in biosimilar entry perpetuate high treatment costs that may reduce therapy adherence and worsen patient outcomes. As biosimilars to other biologics are developed, access will become pressing for more patients. Studies evaluating the real-world effects of policy reforms and treatment costs will be key to guiding future progress.

## Conflicts of interest

None disclosed.

## References

[1] Center for Drug Evaluation and Research Biosimilars action plan. U.S. Food and Drug Administration. https://www.fda.gov/drugs/biosimilars/biosimilars-action-plan.

[2] Cohen H.P., McCabe D. (2020). The importance of countering biosimilar disparagement and misinformation. BioDrugs.

[3] U.S. Food and Drug Administration National drug code directory. https://dps.fda.gov/ndc.

[4] Feng K., Russo M., Maini L., Kesselheim A.S., Rome B.N. (2025). Patient out-of-pocket costs for biologic drugs after biosimilar competition. JAMA Health Forum.

[5] Preserve Access to Affordable Generics and Biosimilars Act. S.142, 118th Cong. https://www.congress.gov/bill/118th-congress/senate-bill/142.

